# Supplementary material for: HSF-1 promotes longevity through ubiquilin-1-dependent mitochondrial network remodelling
Source: Nat Commun. 2024 Nov 12;15:9797. doi: 10.1038/s41467-024-54136-x (PMC11557981; doi:10.1038/s41467-024-54136-x)
Supplement: Supplementary file 1 — Supplementary Information [file 41467_2024_54136_MOESM1_ESM.pdf]

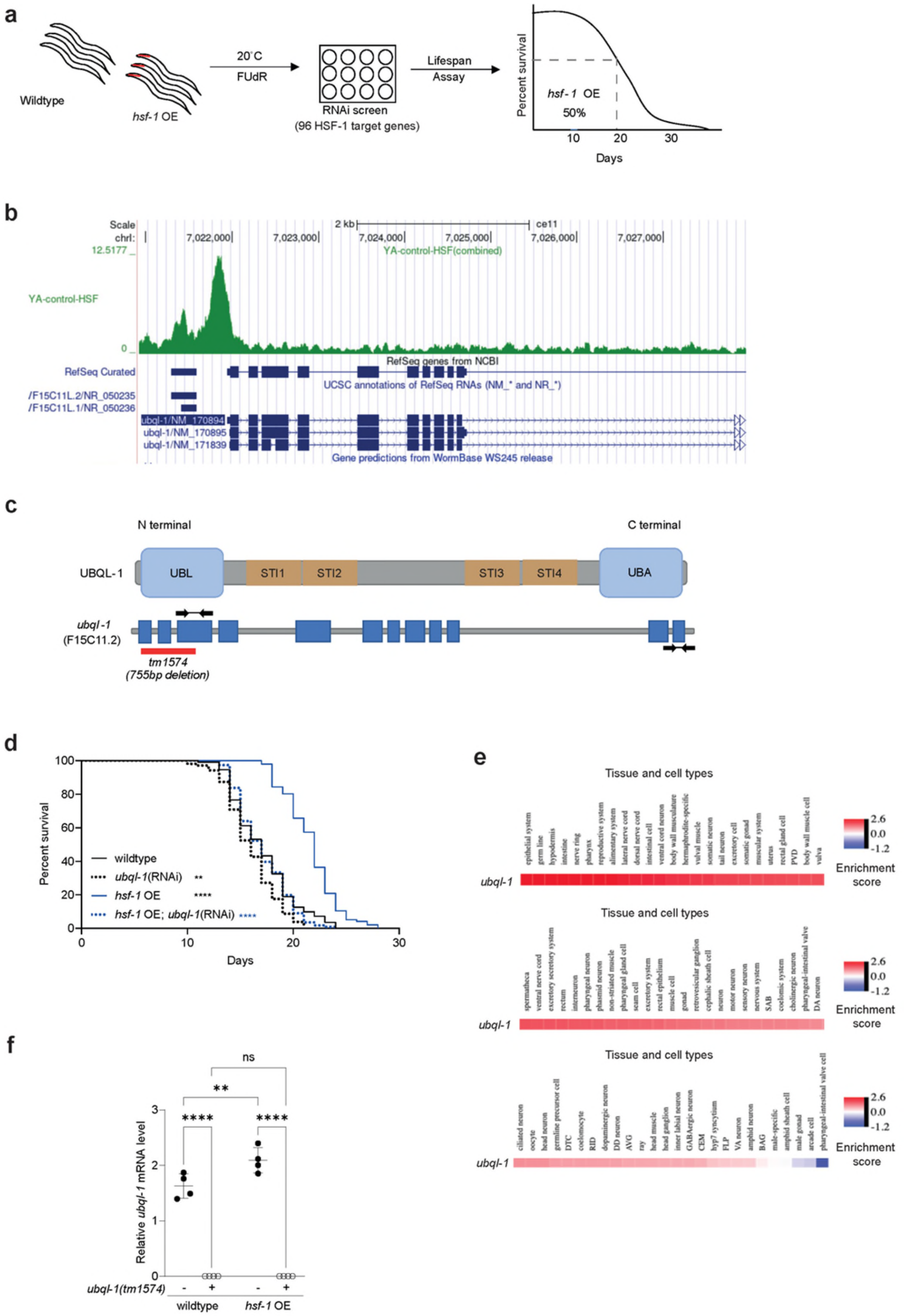

a
Wildtype
hsf-1 OE
20°C
FUdR
RNAi screen
(96 HSF-1 target genes)
Lifespan
Assay
Percent survival
hsf-1 OE
50%
10
20
30
Days
b
Scale
chrI:
2 kb
ce11
7,022,000
7,023,000
7,024,000
7,025,000
7,026,000
7,027,000
12.5177 _
YA-control-HSF(combined)
YA-control-HSF
0 _
RefSeq genes from NCBI
RefSeq Curated
UCSC annotations of RefSeq RNAs (NM_* and NR_*)
/F15C11L.2/NR_050235
/F15C11L.1/NR_050236
ubql-1/NM_170894
ubql-1/NM_170895
ubql-1/NM_171839
Gene predictions from WormBase WS245 release
c
N terminal
C terminal
UBQL-1
UBL
STI1
STI2
STI3
STI4
UBA
ubql-1
(F15C11.2)
tm1574
(755bp deletion)
d
100
80
60
40
20
0
Percent survival
wildtype
ubql-1(RNAi) **
hsf-1 OE ****
hsf-1 OE; ubql-1(RNAi) ****
0
10
20
30
Days
e
Tissue and cell types
epithelial system
germ line
hypodermis
intestine
nerve ring
pharynx
reproductive system
alimentary system
lateral nerve cord
dorsal nerve cord
intestinal cell
ventral cord neuron
body wall musculature
hermaphrodite-specific
vulval muscle
somatic neuron
tail neuron
excretory cell
somatic gonad
muscular system
uterus
rectal gland cell
PVD
body wall muscle cell
vulva
ubql-1
2.6
0.0
-1.2
Enrichment
score
Tissue and cell types
spermatheca
ventral nerve cord
excretory secretory system
rectum
interneuron
pharyngeal neuron
phasmid neuron
non-striated muscle
pharyngeal gland cell
seam cell
excretory system
rectal epithelium
muscle cell
gonad
retrovesicular ganglion
cephalic sheath cell
neuron
motor neuron
sensory neuron
nervous system
SAB
coelomic system
cholinergic neuron
pharyngeal-intestinal valve
DA neuron
ubql-1
2.6
0.0
-1.2
Enrichment
score
Tissue and cell types
ciliated neuron
oocyte
head neuron
germline precursor cell
DTC
coelomocyte
RID
dopaminergic neuron
DD neuron
AVG
ray
head muscle
head ganglion
inner labial neuron
GABAergic neuron
CEM
hyp7 syncytium
FLP
VA neuron
amphid neuron
BAG
male-specific
amphid sheath cell
male gonad
arcade cell
pharyngeal-intestinal valve cell
ubql-1
2.6
0.0
-1.2
Enrichment
score
f
ns
**
****
****
3
2
1
0
Relative ubql-1 mRNA level
ubql-1(tm1574)
-
+
-
+
wildtype
hsf-1 OE

Supplementary Figure 1: *ubql-1* is bound by HSF-1 and broadly expressed **(a)** Schematic representation of RNAi screen to find modifiers of *hsf-1* OE extended survival. **(b)** UCSC genome browser view of *ubql-1* genomic region from HSF-1::GFP ChIP-seq data[16]. **(c)** Schematic diagram of *C. elegans ubql-1* gene and corresponding protein structures. Arrows to the left above exon 3 indicate set of primers used in Supplementary Fig.1f and arrows to the far right above exon 12 indicate primers used in Fig. 1d. **(d)** Lifespan of wildtype and *hsf-1* OE (AM583) animals on empty vector and *ubql-1*(RNAi) (wildtype vs *ubql-1(tm1574),* p=0.0017; wildtype vs *hsf-1*OE, p<0.0001; *hsf-1*OE vs *hsf-1*OE;*ubql-1(tm1574)*, p<0.0001). **(e)** Heatmap depicting tissue/cell-specific expression of *ubql-1*. **(f)** Relative expression of *ubql-1* mRNA on day 1 of adulthood in wildtype, *ubql-1(tm1574)*, *hsf-1* OE, and *hsf-1* OE;*ubql-1(tm1574)* animals grown on OP50 (wildtype vs *ubql-1(tm1574)*, p<0.0001; *hsf-1*OE vs *hsf-1*OE;*ubql-1(tm1574)*, p<0.0001**;** wildtype vs *hsf-1*OE, p=0.0015; *ubql-1(tm1574)* vs *hsf-1*OE;*ubql-1(tm1574),* p>0.9999. Data plotted are the mean +/- SD of 4 biological replicates. All error bars denote SD. Statistical significance was calculated using **(f)** two-way ANOVA with Fishers LSD test or **(d)** Mantel-Cox log rank test. **p < 0.01, ***p < 0.001, ****p<0.0001. Full statistics for lifespan trials (including n values) can be found in Supplementary Data 2. Source data are provided as a Source Data file.

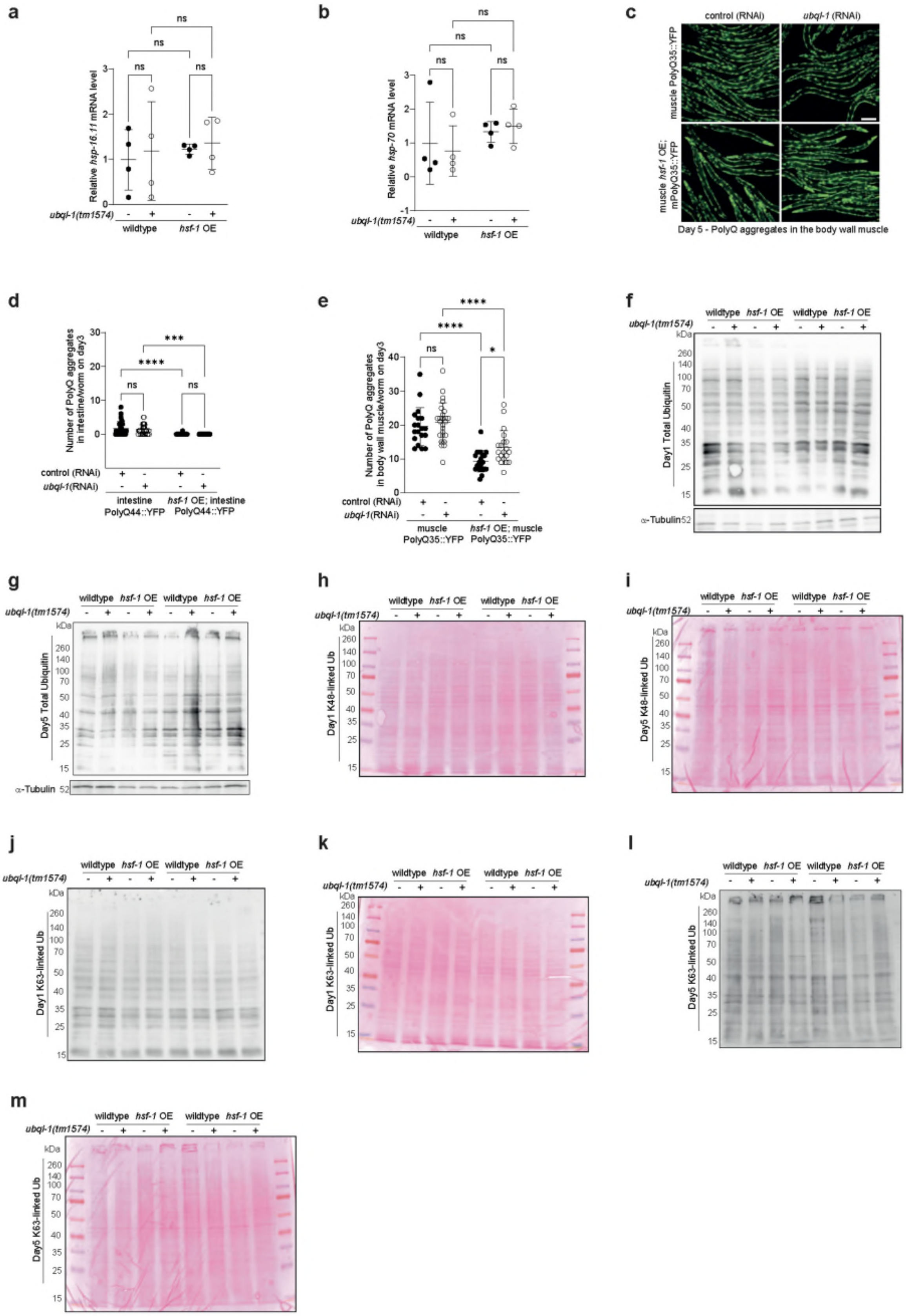
a
ns
ns
ns
ns
Relative hsp-16.11 mRNA level
ubql-1(tm1574)
wildtype
hsf-1 OE
b
ns
ns
ns
ns
Relative hsp-70 mRNA level
ubql-1(tm1574)
wildtype
hsf-1 OE
c
control (RNAi)
ubql-1 (RNAi)
muscle PolyQ35::YFP
muscle hsf-1 OE; mPolyQ35::YFP
Day 5 - PolyQ aggregates in the body wall muscle
d
***
****
ns
ns
Number of PolyQ aggregates in intestine/worm on day3
control (RNAi)
ubql-1(RNAi)
intestine PolyQ44::YFP
hsf-1 OE; intestine PolyQ44::YFP
e
****
****
ns
*
Number of PolyQ aggregates in body wall muscle/worm on day3
control (RNAi)
ubql-1(RNAi)
muscle PolyQ35::YFP
hsf-1 OE; muscle PolyQ35::YFP
f
wildtype
hsf-1 OE
ubql-1(tm1574)
kDa
Day1 Total Ubiquitin
α-Tubulin 52
g
Day5 Total Ubiquitin
α-Tubulin 52
h
Day1 K48-linked Ub
i
Day5 K48-linked Ub
j
Day1 K63-linked Ub
k
Day1 K63-linked Ub
l
Day5 K63-linked Ub
m
Day5 K63-linked Ub

Supplementary Figure 2: *Loss of ubql-1* or overexpression of *hsf-1* does not alter the accumulation of polyubiquitylated or K63-linked ubiquitylated proteins with age

(a and b) Relative expression of *hsp-16.11* and *hsp-70* mRNA on day 1 of adulthood in wildtype, *ubql-1(tm1574)*, *hsf-1* OE or *hsf-1* OE;*ubql-1(tm1574)* animals grown on OP50. Data plotted are the mean +/- SD of 4 biological replicates. Statistical comparisons are **(a)** *hsp-16.11* (wildtype vs *ubql-1(tm1574)*, p=0.7087; *hsf-1*OE vs *hsf-1*OE;*ubql-1(tm1574),* p=0.7936**;** wildtype vs *hsf-1*OE, p=0.6471; *ubql-1(tm1574)* vs *hsf-1*OE;*ubql-1(tm1574),* p=0.7293) and **(b)** *hsp-70 (*wildtype vs *ubql-1(tm1574)*, p=0.6806; *hsf-1*OE vs *hsf-1*OE;*ubql-1(tm1574),* p=0.7648**;** wildtype vs *hsf-1*OE, p=0.5395; *ubql-1(tm1574)* vs *hsf-1*OE;*ubql-1(tm1574)*, p=0.1990). **(c)** Representative images of wildtype and*hsf-1* OE worms expressing body wall muscle polyQ35::YFP grown on empty vector (EV) or *ubql-1*(RNAi) at day 5 of adulthood. Scale bar, 200 µm. **(d and e)** Number of polyglutamine::YFP aggregates present in the **(d)** intestine (Q44::YFP) and **(e)** body wall muscle (Q35::YFP) on day 3 of adulthood in wildtype and *hsf-1* OE animals grown on empty vector and *ubql-1*(RNAi). One of three independent experiments has been shown for intestinal and muscle PolyQ sensors. Statistical comparisons are **(d)** iPolyQ EV (n=30) vs iPolyQ*;ubql*-1(RNAi) (n=30), p=0.2170; *hsf-1*OE;iPolyQ EV (n=32) vs *hsf-1*OE;iPolyQ;*ubql-1*(RNAi) (n=26), p=0.9246; iPolyQ EV vs *hsf-1*OE;ipolyQ EV, p<0.0001; iPolyQ;*ubql*-1(RNAi) vs *hsf*-1OE;iPolyQ;*ubql-1*(RNAi), p=0.0002 and **(e)** mPolyQ EV (n=19) vs mPolyQ*;ubql*-1(RNAi) (n=25), p=0.4250; *hsf-1*OE;mPolyQ EV (n=21) vs *hsf-1*OE;mPolyQ;*ubql-1*(RNAi) (n=20), p=0.0110; mPolyQ EV vs *hsf-1*OE;mPolyQ EV, p<0.0001; mPolyQ;*ubql*-1(RNAi) vs *hsf*-1OE;mPolyQ;*ubql-1*(RNAi), p<0.0001. **(f and g)** SDS-PAGE followed by western blotting and immunodetection for polyubiquitylated proteins or tubulin in wildtype, *ubql-1(tm1574)*, *hsf-1* OE or *hsf-1* OE;*ubql-1(tm1574)* animals on **(f)** day 1 or **(g)** day 5 of adulthood. **(h and i)** Ponceau S staining of western blots used to detect K48-linked ubiquitin on **(h)** day 1 or **(i)** day 5 of adulthood (these panels are an accompaniment to Fig. 2h and i). **(j-m)** SDS-PAGE and western blotting followed by **(j and l)** immunodetection for K63-linked ubiquitylated proteins or **(k and m)** pre-staining of blots with Ponceau S on **(j and k)** day 1 or **(l and m)** day 5 of adulthood. All blots are representative of 4 independent experiments. All error bars denote SD. Statistical significance was calculated using two-way ANOVA with Fishers LSD test. ns, not significant (p>0.05), *p<0.05, **p < 0.01, ***p < 0.001, ****p<0.0001. Source data are provided as a Source Data file.

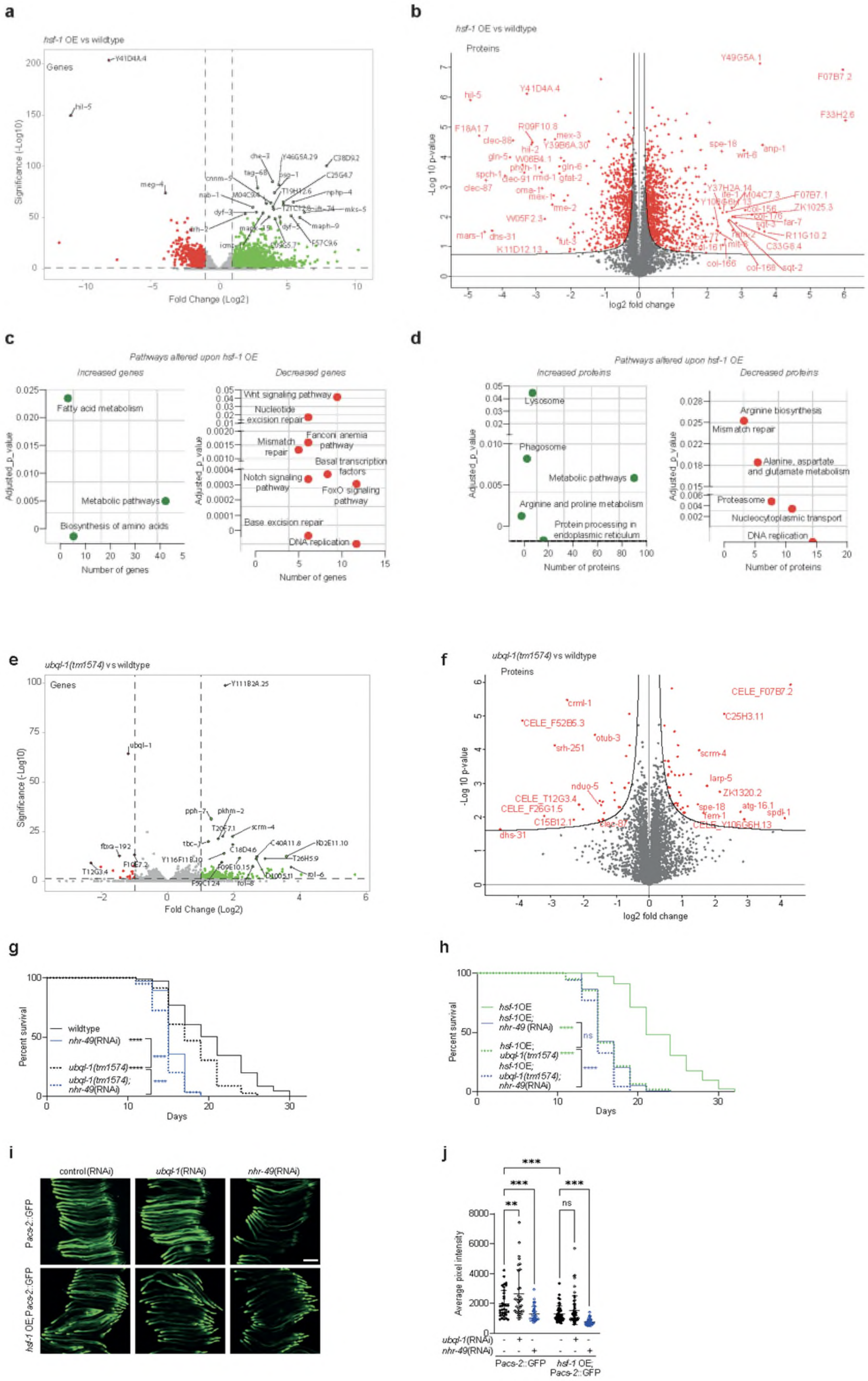

a
hsf-1 OE vs wildtype
Genes
Significance (-Log10)
Fold Change (Log2)
b
hsf-1 OE vs wildtype
Proteins
-Log 10 p-value
log2 fold change
c
Pathways altered upon hsf-1 OE
Increased genes
Decreased genes
Fatty acid metabolism
Metabolic pathways
Biosynthesis of amino acids
Wnt signaling pathway
Nucleotide excision repair
Mismatch repair
Fanconi anemia pathway
Basal transcription factors
Notch signaling pathway
FoxO signaling pathway
Base excision repair
DNA replication
Adjusted_p_value
Number of genes
d
Pathways altered upon hsf-1 OE
Increased proteins
Decreased proteins
Lysosome
Phagosome
Metabolic pathways
Arginine and proline metabolism
Protein processing in endoplasmic reticulum
Arginine biosynthesis
Mismatch repair
Alanine, aspartate and glutamate metabolism
Proteasome
Nucleocytoplasmic transport
DNA replication
Adjusted_p_value
Number of proteins
e
ubql-1(tm1574) vs wildtype
Genes
Significance (-Log10)
Fold Change (Log2)
f
ubql-1(tm1574) vs wildtype
Proteins
-Log 10 p-value
log2 fold change
g
Percent survival
Days
wildtype
nhr-49(RNAi)
ubql-1(tm1574)
ubql-1(tm1574); nhr-49(RNAi)
h
Percent survival
Days
hsf-1OE
hsf-1OE; nhr-49 (RNAi)
hsf-1OE; ubql-1(tm1574)
hsf-1OE; ubql-1(tm1574); nhr-49(RNAi)
ns
i
control(RNAi)
ubql-1(RNAi)
nhr-49(RNAi)
Pacs-2::GFP
hsf-1 OE;Pacs-2::GFP
j
Average pixel intensity
ubql-1(RNAi)
nhr-49(RNAi)
Pacs-2::GFP
hsf-1 OE; Pacs-2::GFP
ns

Supplementary Figure 3: *hsf-1* overexpression leads to changes in genes and proteins associated with metabolic homeostasis

(a and b) Volcano plots showing log2 (fold change, FC) against −log10 (p-value) of **(a)** genes identified from RNASeq analysis or **(b)** proteins identified by label-free quantitative mass-spectrometry of *hsf-1* OE *vs* wildtype worms. **(c and d)** KEGG pathway enrichment analysis of significantly up- and downregulated (right) **(c)** genes and **(d)** proteins upon *hsf-1* overexpression. **(e and f)** Volcano plots showing log2 (fold change, FC) against −log10 (q-value) of **(e)** genes identified from RNASeq analysis or **(f)** proteins identified by label-free quantitative mass-spectrometry of *ubql-1(tm1574) vs* wildtype worms, **(g and h)** Lifespan analysis of **(g)** wildtype and *ubql-1 (tm1574)* mutants subjected to *nhr-49* RNAi (wildtype vs *ubql-1(tm1574)*, p<0.0001; wildtype vs wildtype;*nhr-49*(RNAi), p<0.0001; *ubql-1(tm1574)* vs *ubql-1(tm1574)*;*nhr-49*(RNAi), p<0.0001; wildtype;*nhr-49*(RNAi) vs *ubql-1(tm1574)*, p<0.0001) and **(h)** *hsf-1* OE and *hsf-1* OE;*ubql-1(tm1574)* animals subjected to *nhr-49* RNAi (*hsf-1*OE vs h*sf-1*OE;*ubql-1(tm1574)*, p<0.0001; *hsf-1*OE vs *hsf-1*OE;*nhr-4*9(RNAi), p<0.0001; *hsf-1*OE;*ubql-1(tm1574)* vs *hsf-1*OE;*ubql-1(tm1574);nhr-49*(RNAi), p=0.0010; *hsf-1*OE;*nhr-4*9(RNAi) vs *hsf-1*OE;*ubql-1(tm1574),* p=0.9438). **(i)** Representative fluorescence images of wildtype, or *hsf-1* OE, NHR-49 reporter animals (*acs-2*p::GFP) on day 1 of adulthood following exposure to empty vector (EV) control, *ubql-1*(RNAi) or *nhr-49*(RNAi). Scale bar, 200 μm. **(j)** Quantification of *acs-2*p::GFP fluorescence on day 1 of adulthood in wildtype, or *hsf-1* OE worms grown on empty vector control, *ubql-1*(RNAi) or *nhr-49*(RNAi). Data are represented as mean +/- SD (*acs-2*p::GFP EV (n=35) vs *acs-2*p::GFP;*ubql-1*(RNAi) (n=33), p=0.0011; *acs-2*p::GFP EV vs *acs-2*p::GFP;*nhr-49*(RNAi) (n=38), p=0.0009; *acs-2*p::GFP EV vs *hsf-1*OE;*acs-2*p::GFP EV (n=50), p=0.0006; *hsf-1*OE;*acs-2*p::GFP EV vs *hsf-1*OE;*acs-2*p::GFP;*ubql-1*(RNAi) (n=55), p=0.1854; *hsf-1*OE;*acs-2*p::GFP EV vs *hsf-1*OE;*acs-2*p::GFP;*nhr-49*(RNAi) (n=54), p=0.0009). Statistical significance was calculated by **(g and h)** Mantel-Cox log rank test and **(j)** two-way ANOVA followed by Fishers LSD test. ns, not significant (p>0.05), ****p<0.0001. Full statistics for lifespan trials (including n values) can be found in Supplementary Data 2. Source data are provided as a Source Data file.

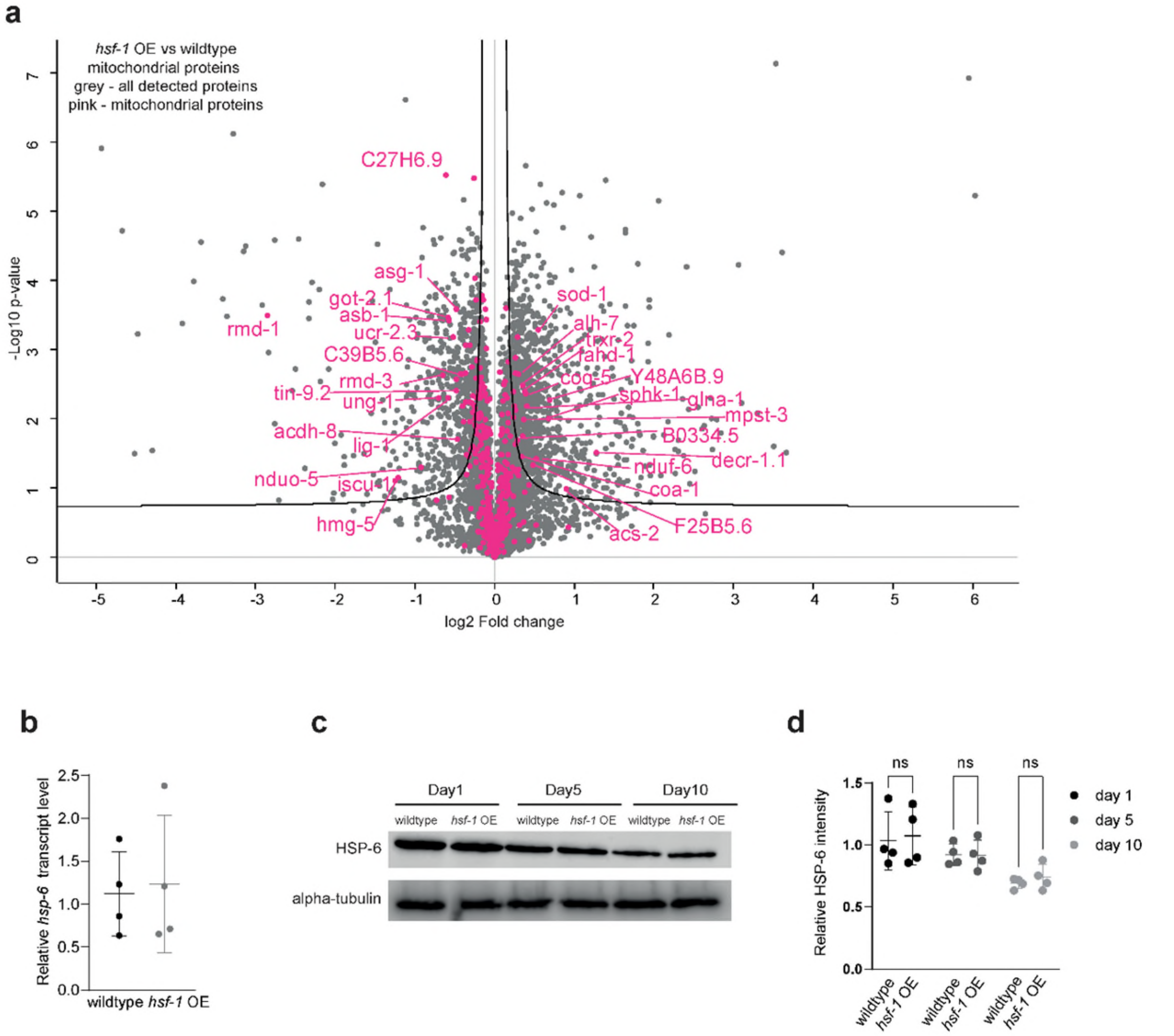
a
hsf-1 OE vs wildtype
mitochondrial proteins
grey - all detected proteins
pink - mitochondrial proteins
C27H6.9
asg-1
got-2.1
asb-1
ucr-2.3
rmd-1
C39B5.6
rmd-3
tin-9.2
ung-1
acdh-8
lig-1
nduo-5
iscu-1
hmg-5
sod-1
alh-7
trxr-2
fahd-1
coq-5
Y48A6B.9
sphk-1
glna-1
mpst-3
B0334.5
decr-1.1
nduf-6
coa-1
F25B5.6
acs-2
-Log10 p-value
log2 Fold change
b
Relative hsp-6 transcript level
wildtype
hsf-1 OE
c
Day1
Day5
Day10
wildtype
hsf-1 OE
HSP-6
alpha-tubulin
d
ns
Relative HSP-6 intensity
day 1
day 5
day 10
wildtype
hsf-1 OE

Supplementary Figure 4: *hsf-1* overexpression alters the abundance of a sub-set of the mitochondrial proteome but does not activate the UPR^{mt}

(a) Volcano plot showing log2 (fold change, FC) against −log10 (p-value) of mitochondrial proteins detected in *hsf-1* OE and wildtype worms by mass-spectrometry. Pink dots mark all detected mitochondrial proteins, with significantly altered mitochondrial proteins outside the black curve (FDR < 0.05). **(b)** Relative expression of the canonical UPR^{mt} gene *hsp-6* in wildtype and *hsf-1*OE worms. Data plotted are the mean +/- SD of 4 biological replicates and values were normalized to the geometric mean of the housekeeping genes *rpb-2*, *pmp-3* and *cdc-42* (unpaired, two-tailed Student's t-test, p=0.8120) **(c)** Representative western blot following immunodetection for HSP-6 or alpha-tubulin in wildtype and *hsf-1* OE protein extracts on day 1, 5, and 10 of adulthood. Blots presented are representative of 4 experiments. **(d)** Quantification of HSP-6 levels relative to tubulin in wildtype and *hsf-1* OE worms on day 1, 5, and 10 of adulthood. Data plotted are the mean +/- SD of four biological replicates. (day 1 wildtype vs *hsf-1*OE, p =0.7158; day5 wildtype vs *hsf-1*OE, p=0.9673; day10 wildtype vs *hsf-1*OE, p=0.6669). Statistical significance was calculated by two-way ANOVA followed by Fishers LSD test. ns, not significant (p>0.05). Source data are provided as a Source Data file.

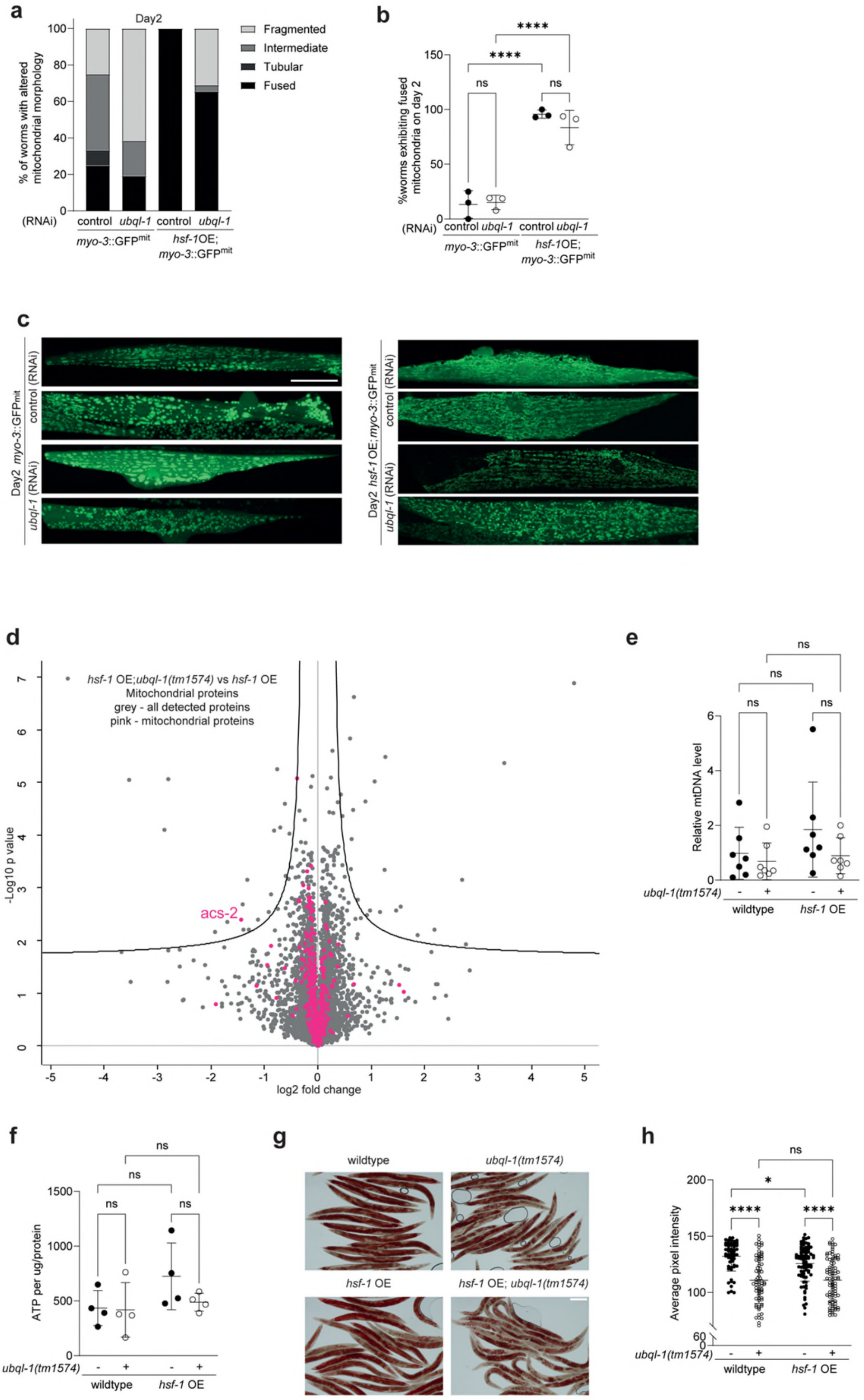
a
Day2
% of worms with altered mitochondrial morphology
100
80
60
40
20
0
Fragmented
Intermediate
Tubular
Fused
(RNAi)
control
ubql-1
control
ubql-1
myo-3::GFPmit
hsf-1OE; myo-3::GFPmit
b
%worms exhibiting fused mitochondria on day 2
150
100
50
0
****
****
ns
ns
(RNAi)
control
ubql-1
control
ubql-1
myo-3::GFPmit
hsf-1OE; myo-3::GFPmit
c
Day2 myo-3::GFPmit
control (RNAi)
ubql-1 (RNAi)
Day2 hsf-1 OE; myo-3::GFPmit
control (RNAi)
ubql-1 (RNAi)
d
hsf-1 OE;ubql-1(tm1574) vs hsf-1 OE
Mitochondrial proteins
grey - all detected proteins
pink - mitochondrial proteins
acs-2
-Log10 p value
log2 fold change
-5
-4
-3
-2
-1
0
1
2
3
4
5
7
6
5
4
3
2
1
0
e
Relative mtDNA level
6
4
2
0
ns
ns
ns
ns
ubql-1(tm1574)
-
+
-
+
wildtype
hsf-1 OE
f
ATP per ug/protein
1500
1000
500
0
ns
ns
ns
ns
ubql-1(tm1574)
-
+
-
+
wildtype
hsf-1 OE
g
wildtype
ubql-1(tm1574)
hsf-1 OE
hsf-1 OE; ubql-1(tm1574)
h
Average pixel intensity
200
150
100
60
0
ns
*
****
****
ubql-1(tm1574)
-
+
-
+
wildtype
hsf-1 OE

Supplementary Figure 5: *Ubql-1* regulates total fat levels

(a) Proportion of mitochondrial morphologies observed in $p_{myo\text{-}3}$::GFP(mit) worms and *hsf-1* OE; $p_{myo\text{-}3}$::GFP(mit) worms grown on empty vector control or *ubql-1*(RNAi) on day 2 adulthood (*myo-3*::GFP(mit), n=12; *myo-3*::GFP(mit);*ubql-1*(RNAi), n=26; *hsf-1*OE; *myo-3*::GFP(mit), n= 20; *hsf-1*OE; *myo-3*::GFP(mit);*ubql-1*(RNAi), n=29. **(b)** Prevalence of fused mitochondria in muscle tissues of wildtype and *hsf-1* OE worms, +/- *ubql-1* (RNAi), on day 2 of adulthood. Values are the mean +/-SD of three independent experiments (*myo-3*::GFP(mit) vs *myo-3*::GFP(mit);*ubql-1*(RNAi), p=0.8344; *hsf-1*OE; *myo-3*::GFP(mit) vs *hsf-1*OE; *myo-3*::GFP(mit);*ubql-1*(RNAi), p=0.1994; *myo-3*::GFP(mit) vs *hsf-1*OE;*myo-3*::GFP(mit), p<0.0001; *myo-3*::GFP(mit);*ubql-1*(RNAi) vs *hsf-1*OE;*myo-3*::GFP(mit);*ubql-1*(RNAi), p<0.0001). **(c)** Representative confocal microscope images of *myo-3*p::GFPmit within muscle tissues of wildtype or *hsf-1* OE worms +/- *ubql-1*(RNAi) on day 2 of adulthood. Scale bar, 20 µm. **(d)** Volcano plot showing log2 (fold change, FC) against −log10 (q-value) of mitochondrial proteins detected in *hsf-1* OE and *hsf-1* OE;*ubql-1(tm1574)* worms by mass-spectrometry. Pink dots mark all detected mitochondrial proteins, with significantly altered proteins outside the black curve (FDR < 0.05). **(e)** Levels of mtDNA (*ND1*) relative to genomic DNA (*cdc-42*) as detected by real-time quantitative PCR in wildtype, *ubql-1(tm1574)*, *hsf-1* OE and *hsf-1* OE; *ubql-1(tm1574)* worms on day 1 of adulthood following growth on OP50 bacteria. Data plotted are the mean +/- SD of 7 biological replicates (wildtype vs *ubql-1(tm1574),* p=0.6171; *hsf-1*OE vs *hsf-1*OE;*ubql-1(tm1574),* p=0.1148**;** wildtype vs *hsf-1*OE, p=0.1509; *ubql-1(tm1574)* vs *hsf-1*OE;*ubql-1(tm1574),* p=0.7265). **(f)** ATP levels in wildtype, *ubql-1(tm1574)*, *hsf-1* OE and *hsf-*1 OE; *ubql-1(tm1574)* animals at day 1 of adulthood. Data are the mean +/- SD of 4 biological replicates (wildtype vs *ubql-1(tm1574),* p=0.9171; *hsf-1*OE vs *hsf-1*OE;*ubql-1(tm1574),* p=0.1482**;** wildtype vs *hsf-1*OE, p=0.0821; *ubql-1(tm1574)* vs *hsf-1*OE;*ubql-1(tm1574),* p=0.6550). **(g** and **h)** Triglyceride levels in day 5 wildtype, *ubql-1(tm1574)*, *hsf-1* OE and *hsf-1* OE;*ubql-1(tm1574)* adult animals. **(g)** Representative images and **(h)** quantification representing the relative lipid content in whole body of worms. (wildtype (n=58) vs *ubql-1(tm1574)* (n=71), p<0.0001; *hsf-1*OE (n=86) vs *hsf-1*OE;*ubql-1(tm1574)* (n=87), p<0.0001**;** wildtype vs *hsf-1*OE, p=0.0307; *ubql-1(tm1574)* vs *hsf-1*OE;*ubql-1(tm1574),* p=0.9626). One of three independent experiments has been shown. All error bars denote SD. Scale bar, 500µm. Statistical significance was calculated by **(b, e, f, and h)** two-Way ANOVA with Fishers LSD test ns, not significant (p>0.05), *p<0.05, **p < 0.01, ****p<0.0001. Source data are provided as a Source Data file.

Target Gene	Forward sequence	Reverse sequence
ubql-1 (Exon 3)	TCTCACACAGCACAAAATCGC	CCTCCCATTGTTGGTGCAGA
ubql-1 (Exon 12)	GGGAGGAGGAAGACCCTCAT	TTCTGGCACGATCCGAGAAG
hsp-16.11	TGGCTCAGATGGAACGTCAA	TGGCTTGAACTGCGAGACAT
hsp-70 (C12C8.1)	CTACATGCAAAGCGATTGGA	GGCGTAGTCTTGTTCCCTTC
hsp-6	GTTATCGAGAACGCAGAAGGAG	CATCCTTAGTAGCTTGACGCTG
pmp-3	GTTCCCGTGTTCATCACTCAT	ACACCGTCGAGAAGCTGTAGA
rpb-2	AACTGGTATTGTGGATCAGGTG	TTTGACCGTGTCGAGATGC
cdc-42	TCGACAATTACGCCGTCACA	GAAACACGTCGGTCTGTGGA
F44E5.4	GTTGAGATCCTCGCCAACTC	GCTGCATCTCCAACCAATCT
nd-1 (genomic)	AGCGTCATTTATTGGGAAGAAGAC	AAGCTTGTGCTAATCCCATAAATGT
cdc-42 (genomic)	ATGGTAAAGAAACGCTCGTG	TGAAAAATACGGATGAGTCACA

Supplementary Table 1. List of primers used in this study.
